# Rab40c regulates focal adhesions and PP6 activity by controlling ANKRD28 ubiquitylation

**DOI:** 10.26508/lsa.202101346

**Published:** 2022-05-05

**Authors:** Ke-Jun Han, Valeryia Mikalayeva, Scott A Gerber, Arminja N Kettenbach, Vytenis A Skeberdis, Rytis Prekeris

**Affiliations:** 1 Department of Cell and Developmental Biology, University of Colorado Anschutz Medical Campus, Aurora, CO, USA; 2 Institute of Cardiology, Lithuanian University of Health Sciences, Kaunas, Lithuania; 3 Department of Molecular and Systems Biology, Geisel School of Medicine at Dartmouth, Hanover, NH, USA; 4 Department of Biochemistry and Cell Biology, Geisel School of Medicine at Dartmouth, Hanover, NH, USA; 5 Norris Cotton Cancer Center, Lebanon, NH, USA

## Abstract

Rab40c is a SOCS box–containing protein which binds Cullin5 to form a ubiquitin E3 ligase complex (Rab40c/CRL5) to regulate protein ubiquitylation. However, the exact functions of Rab40c remain to be determined, and what proteins are the targets of Rab40c-Cullin5–mediated ubiquitylation in mammalian cells are unknown. Here we showed that in migrating MDA-MB-231 cells Rab40c regulates focal adhesion’s number, size, and distribution. Mechanistically, we found that Rab40c binds the protein phosphatase 6 (PP6) complex and ubiquitylates one of its subunits, ankyrin repeat domain 28 (ANKRD28), thus leading to its lysosomal degradation. Furthermore, we identified that phosphorylation of FAK and MOB1 is decreased in Rab40c knock-out cells, which may contribute to focal adhesion site regulation by Rab40c. Thus, we propose a model where Rab40c/CRL5 regulates ANKRD28 ubiquitylation and degradation, leading to a decrease in PP6 activity, which ultimately affects FAK and Hippo pathway signaling to alter focal adhesion dynamics.

## Abstract (precis)

The role of novel Rab40c/CRL5 ubiquitylation complex in regulating PP6 activity and cell migration.

## Introduction

Cell migration is a fundamental cellular function that is involved in many important biological processes, including embryological development, tissue formation, wound healing, and cancer metastasis. In response to extracellular and intracellular signals, migratory cells reorganize their cytoskeleton and endocytic transport to form actin-dependent migratory cell protrusions, such as lamellipodia, and establish a front-to-rear polarity (Maritzen et al, 2015; Seetharaman & Etienne-Manneville, 2020; Shellard & Mayor, 2020; SenGupta et al, 2021). By interaction with the ECM, cells can form integrin-based macromolecular adhesive structures called focal adhesions (FAs) (Burridge & Guilluy, 2016; Burridge, 2017; Legerstee & Houtsmuller, 2021; Mishra & Manavathi, 2021). FAs include numerous scaffoldings and signaling proteins, such as talin, vinculin, zyxin, paxillin, p130Cas, and α-actinin, that regulate FA formation and disassembly during cell migration (Stutchbury et al, 2017; Martinez & Rainero, 2019; Ibata & Terentjev, 2020). One of the main functions of FAs is to physically connect the cellular actin cytoskeleton to ECM, therefore sensing, integrating, and transducing extracellular signaling. In addition, FAs can serve as anchor points to generate tensional forces to push cells forward during cell migration (Parsons et al, 2010; Burridge & Guilluy, 2016; Yamada et al, 2019; Seetharaman & Etienne-Manneville, 2020).

FAs formation is initiated by ECM binding to cell surface receptors, primarily integrins, at the leading edge (Legerstee & Houtsmuller, 2021; Schumacher et al, 2021). Newly formed nascent adhesions gradually grow and change their protein composition to mature into FAs. FAs usually localize at the cell periphery, where they associate with the ends of stress fibers (Zaidel-Bar et al, 2004; Burridge & Guilluy, 2016). With nascent adhesion formation at the leading edge, the FAs at the cell rear need to be disassembled to promote rear end retraction and efficient cell migration. Therefore, FAs are highly dynamic, and their number, size, and distribution can rapidly change in response to internal or external signals. It is well established that the dynamic process of FAs is under the regulation of protein tyrosine kinases such as FAK and small GTPases of the Rho/Rac family (Nobes & Hall, 1999; Katoh, 2017; Martínez et al, 2020). Recently, Hippo signaling pathways also have been suggested to regulate cell migration by controlling FA dynamics and mediating mechano-sensing of changes in ECM stiffness and composition (Nardone et al, 2017; Rausch & Hansen, 2020; Wang et al, 2021). Although extensively studied, our current understanding of the molecular mechanisms underlying FA dynamics is still limited.

Rab GTPases are key regulators of membrane trafficking and play an important role in cell migration. We previously demonstrated that the Rab40 subfamily of small GTPases is required for breast cancer cell invasion by promoting ECM degradation and invadopodia formation (Jacob et al, 2013; Jacob et al, 2016; Linklater et al, 2021), although it remains to be fully understood how proteins within the Rab40 family function and what molecular machinery is governing Rab40-dependent cell migration and invasion. Rab40 is a unique subfamily of small monomeric GTPases that include four closely related proteins: Rab40a, Rab40al, Rab40b, and Rab40c, and is characterized by the presence of suppressors of the cytokine signaling (SOCS) box at their C-terminal (Pereira-Leal & Seabra, 2000; Klöpper et al, 2012; Homma et al, 2021). The SOCS box in other proteins has been shown to bind Cullin5–Elongin B/C to form ubiquitin E3 ligase complex (CRL5), used to mediate target protein ubiquitination and degradation (Kile et al, 2002; Linossi & Nicholson, 2012), and we recently demonstrated that Rab40b binds CRL5 to ubiquitylate EPLIN and Rap2, thus promoting cell migration by altering stress fiber formation and leading edge actin dynamics (Linklater et al, 2021). Furthermore, Rab40a was implicated in mediating proteasomal degradation of RhoU, thus regulating FA dynamics (Dart et al, 2015). All these findings suggest that the Rab40 subfamily of small monomeric GTPases may have evolved to regulate actin dynamics and FA turn-over by mediating ubiquitylation and degradation of a specific subset of proteins that regulate cell migration.

In this study, we focus on investigating the functions of Rab40c because it remains to be determined whether Rab40c regulates FA dynamics. In addition, it remains unclear what are the targets of Rab40c-dependent ubiquitylation and degradation are. Here, we show that Rab40c regulates the size, location, and number of FAs in breast cancer cells, while also demonstrating that Rab40c interacts with ankyrin repeat domain 28 protein (ANKRD28), which is a scaffolding subunit of heterotrimeric protein phosphatase PP6 complex. Importantly, ANKRD28-containing PP6 complex inhibits FA formation, and Rab40c directly regulates ubiquitination and degradation of ANKRD28 in breast cancer cells. Finally, we found that Rab40c regulates the Hippo signaling pathway, possibly through MOB1 dephosphorylation by an ANKRD28-containing PP6 subcomplex. Based on all these data, we propose that Rab40c/CRL5 contributes to regulation of FAs by inhibiting the formation and activity of ANKRD28-containing PP6 subcomplexes, which in turn regulates Hippo signaling and mechanosensing.

## Results

### Rab40c regulates FA formation

**Figure 1.**
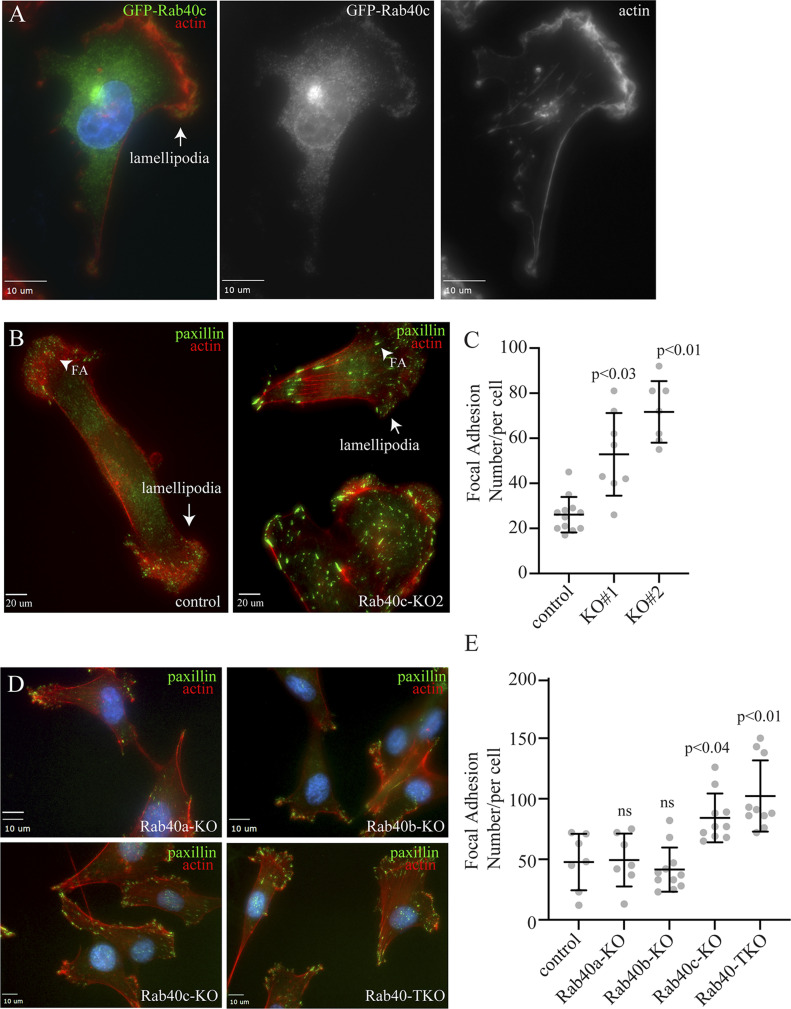
Rab40c regulates focal adhesions. **(A)** MDA-MB-231 cells stably expressing GFP-Rab40c were plated on collagen-coated coverslips and then fixed and stained with phalloidin-Alexa Fluor 594. Arrow points to the lamellipodia. **(B)** Control or Rab40c-KO MDA-MB-231 cells fixed and stained with phalloidin-Alexa Fluor 594 (red) and anti-paxillin antibodies (green). Arrows point to the lamellipodia, and arrowheads point to FAs. **(C)** Quantification of number of FAs per cell for control and Rab40c KO cells. n ≥ 10 cells per condition. Data shown are means and SDs derived from two independent experiments. **(D)** Control, Rab40a-KO, Rab40b-KO, Rab40c-KO, and Rab40a/b/c-KO (Rab40-TKO) MDA-MB-231 cells were fixed and stained with phalloidin-Alexa Fluor 594 (red) and anti-paxillin antibodies (green). **(E)** Quantification of number of FAs per cell for control, Rab40a-KO, Rab40b-KO, Rab40c-KO, and Rab40-TKO cells. n ≥ 10 cells per condition. Data shown are means and SDs derived from two independent experiments. Source Data for Fig 1 shows all uncropped and unmodified Western blots used in the Figs 1–3. Source data are available for this figure.

**Figure S1.**
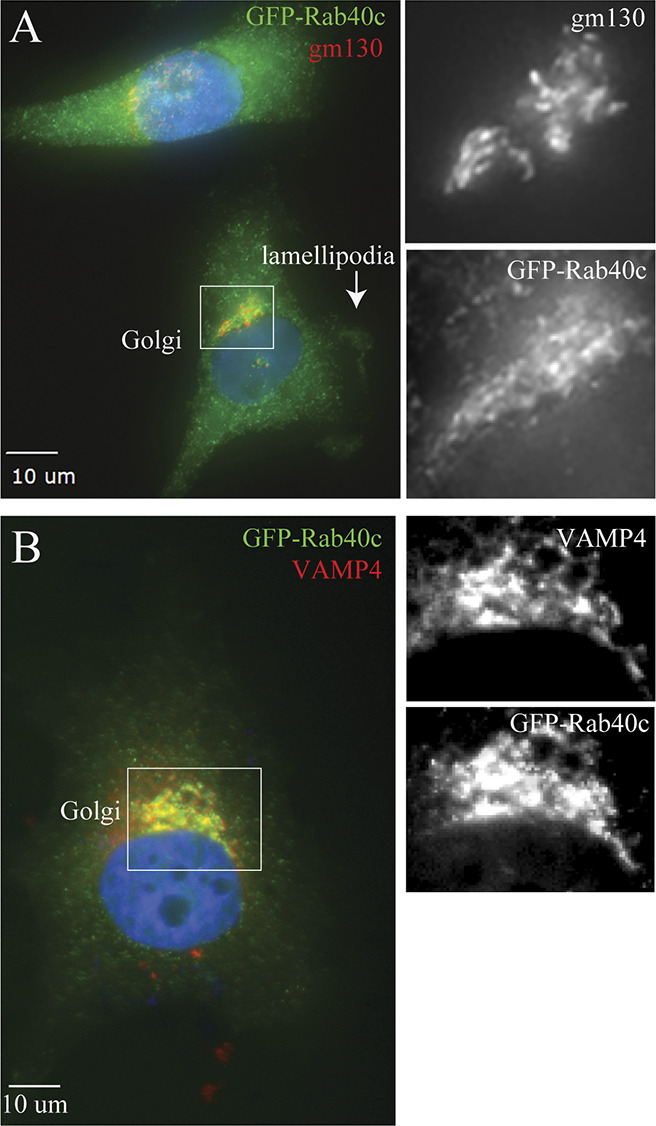
Rab40c localization in MDA-MB-231 cells. **(A, B)** MDA-MB-231 cells stably expressing GFP-Rab40c were plated on collagen-coated coverslips and then stained with anti-GM130 (A; red) or anti-VAMP4 (B; red) antibodies. Boxes mark the region of interest shown in the inset. Arrow points to the lamellipodia.

Rab40b small monomeric GTPases have recently emerged as regulators of localized ubiquitylation of several proteins involved in mediating cell migration (Linklater et al, 2021). Rab40c is another member of the Rab40 subfamily of proteins that is defined by the presence of SOCS box and their ability to mediate Cullin5-dependent protein ubiquitylation. However, despite its close similarity to Rab40b (Duncan et al, 2021), it remains unclear whether Rab40c plays any role in regulating cell migration, and whether Rab40c and Rab40b have overlapping functions. Because Rab GTPases are key regulators of intracellular membrane trafficking and localization of different membrane compartments, we first decided to examine the subcellular localization of human Rab40c. To that end, we created a MDA-MB-231 cell line stably expressing GFP-tagged human Rab40c, and then analyzed the distribution of GFP-Rab40c by immunofluorescence microscopy. As shown in Fig 1A, although most GFP-Rab40c is present in the cytosol, a subpopulation of GFP-Rab40c can clearly be observed at the front edge of the lamellipodia where it colocalizes with actin ruffles. GFP-Rab40c also accumulates and colocalizes with GM130 and VAMP4 (Fig S1), two Golgi markers, suggesting that in addition to potentially regulating lamellipodia dynamics, GFP-Rab40c may also regulate protein transport from Golgi to the plasma membrane.

**Figure S2.**
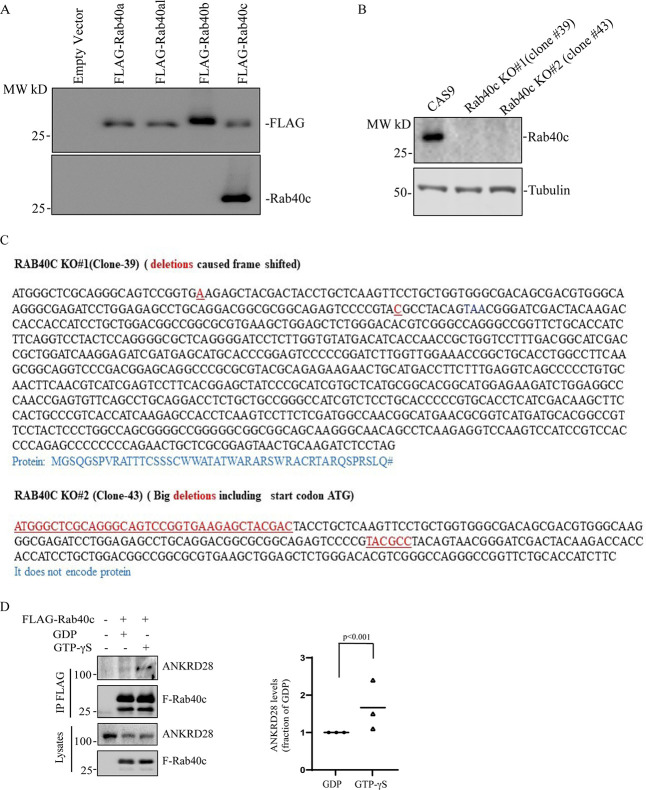
Generation of Rab40c Knock Out MDA-MB-231 cell lines. **(A)** Testing specificity of anti-Rab40c antibody. 293T cells were transfected with empty vector, FLAG-Rab40a, FLAG-Rab40al, FLAG-Rab40b, or FLAG-Rab40c. Cell lysates were then blotted with anti-FLAG or anti-Rab40c antibodies. **(B)** Immunoblotting of cell lysates from control and Rab40c-KO cells with anti-Rab40c and anti-tubulin antibodies. **(C)** Genotyping two of Rab40c-KO MDA-MB-231 cell lines. Deletions are highlight in red. Predicted amino acids are shown under the nucleotide sequences. **(D)** GTP-dependency of Rab40c interaction with ANKRD28. Lysates from MDA-MB-231 cells expressing FLAG-Rab40c were incubated with anti-FLAG antibodies in the presence of either GDP or GTPγS. FLAG-Rab40c was then immunoprecipitated and blotted with ANKRD28 antibodies. The data shown are the means and SEM derived from three independent experiments.

Our previous study showed that knock-out of all three Rab40 isoforms (Rab40a, Rab40b, and Rab40c) inhibited cell migration in part by increasing the number and size of focal adhesion (FA) sites (Linklater et al, 2021). What remains unclear is which Rab40 sub-family members may be involved in directly regulating FA dynamics. To test whether Rab40c has effects on FAs, we generated Rab40c knockout cell lines by CRISPR/Cas-mediated genome editing, which have been validated by genomic sequencing and Western blot (Fig S2B and C). These cells were then stained with an anti-paxillin antibody to assess the steady-state number and distribution of FAs. As previously reported in the control cells, paxillin-positive dot-like FAs were mostly present at the cell periphery, especially leading-edge lamellipodia (Fig 1B). In contrast, within Rab40c-depleted cells, an increased quantity with larger paxillin-positive FAs was observed (Fig 1B and C). Importantly, FAs were not limited to the periphery of the cell, but instead were scattered throughout the plasma membrane (Fig 1B). Quantification of the number of FAs per cell shows a significant increase in FAs in Rab40c KO cells (Fig 1C). This change seems to be mediated predominately by Rab40c-KO because neither Rab40a nor Rab40b KOs alone led to an increase in FA number and size (Fig 1D and E). Consistent with this and with our previously published data (Linklater et al, 2021), knocking-out all three Rab40 isoforms (Rab40a, Rab40b, and Rab40c; Rab40-TKO) did not further increase the number of FAs as compared with Rab40c-KO (Fig 1D and E).

**Figure 2.**
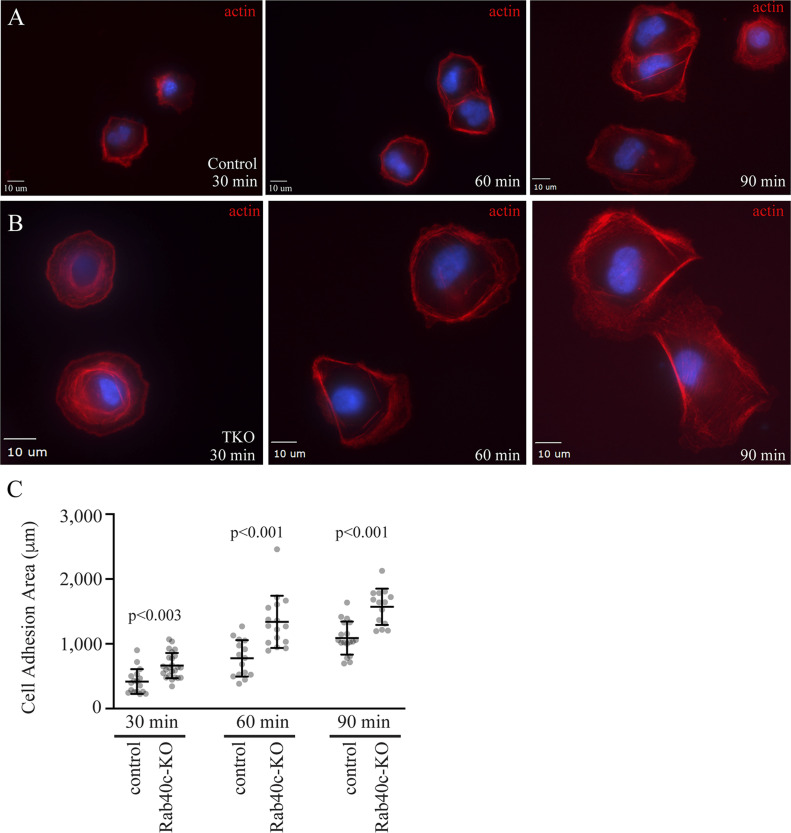
Rab40c regulates cell-ECM adhesion. **(A, B)** Control or Rab40c-KO MDA-MB-231 cells were plated on collaged-coated coverslips and incubated for 30, 60, and 90 min. Cells were than fixed and stained with phalloidin-Alexa Fluor 594. **(C)** Quantification of number of adhesion area per cell for control and Rab40c KO cells. n ≥ 10 cells per condition. Data shown are means and SDs derived from three independent experiments. Source data for Fig 2 shows all uncropped and unmodified Western blots used in Figs 4–6. Source data are available for this figure.

To further test the effect of Rab40c-KO on cell-ECM adhesion, we performed adhesion assays. Cells were plated on collagen-coated coverslips and incubated for 30, 60, or 90 min. The surface area of cell-ECM adhesion was then measured and compared between control and Rab40c-KO cells. As shown in Fig 2A–C, Rab40c depletion increased the spread of the cells on collagen substrate, consistent with the increase in number and size of FAs in Rab40c-KO cells.

### Identification of Rab40-interacting proteins

**Figure 3.**
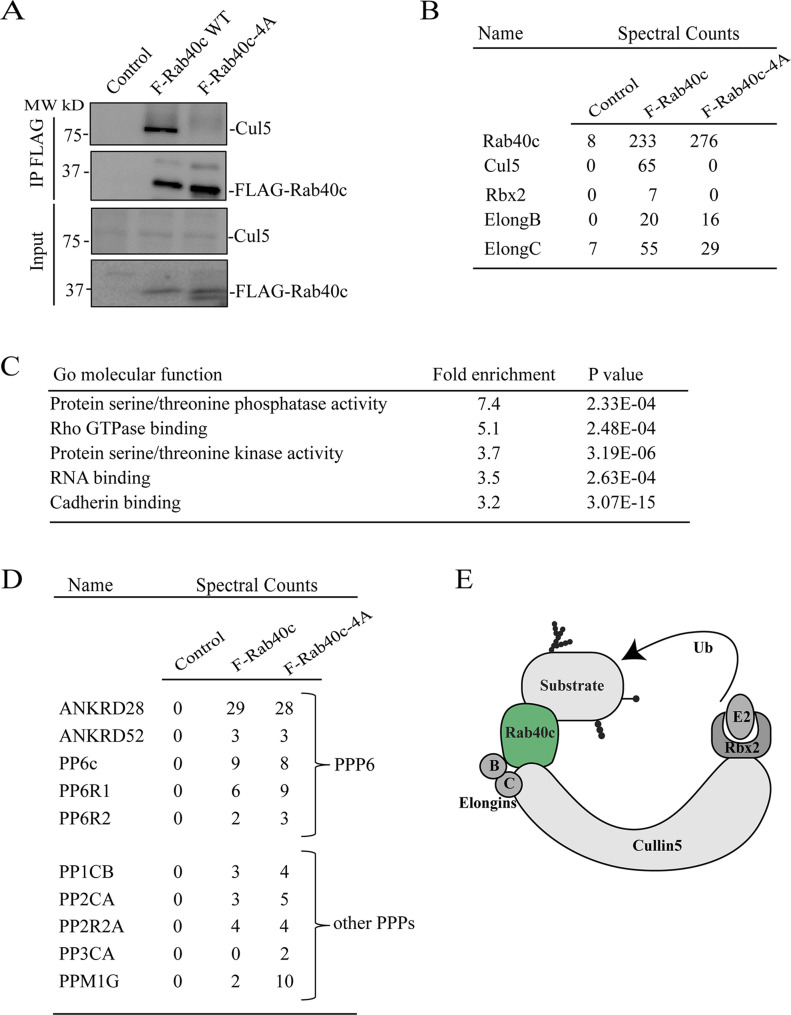
Identification of Rab40c-interacting proteins. **(A)** Cell lysates from control, FLAG-Rab40c and FLAG-Rab40c-4A expressing cells were immunoprecipitated with anti-FLAG antibody. Immunoprecipitates and cell lysates then were then blotted with anti-FLAG or anti-Cul5 antibodies. **(B)** The list of Cul5 ligase complex (CRL5) subunits identified by mass spectrometry from FLAG-Rab40c and FLAG-Rab40c-4A–expressing cells. **(C)** Gene Ontology enrichment analysis of proteins identified by mass spectrometry from FLAG-Rab40c and FLAG-Rab40c-4A immunoprecipitates. **(D)** List of phosphoprotein phosphatases identified by mass spectrometry from FLAG-Rab40c and FLAG-Rab40c-4A immunoprecipitates. **(E)** A model showing Rab40c-CRL5 E3 ligase complex. Ub, ubiquitin.

To understand how Rab40c contributes to regulation of FAs, we next sought to identify Rab40c-interacting partners. Rab40c has a SOCS box at its C terminus, and it is well established that a highly conserved LPLP motif in the SOCS box is necessary for the binding to Cullin5 (Cul5). To examine whether this motif in Rab40c is also important for the binding to Cul5, we mutated the LPLP sequence to AAAA (FLAG-Rab40c-4A) and established MDA-MB-231 cell lines stably expressing either FLAG-Rab40c or FLAG-Rab40c-4A. Then, FLAG tagged proteins were immunoprecipitated with an anti-FLAG antibody. As shown in Fig 3A, endogenous Cul5 co-precipitated with FLAG-Rab40c. However, FLAG-Rab40c-4A has lost its ability to bind Cul5, confirming that the LPLP motif mediates Rab40c binding to Cul5.

To identify proteins that bind to Rab40c, we co-immunoprecipitated FLAG-Rab40c with either anti-FLAG antibody–conjugated beads or control mice IgG beads, followed by analysis using mass spectrometry. As expected, Cul5 and Rbx2 both co-immunoprecipitated with FLAG-Rab40c, but not IgG control or FLAG-Rab40c-4A (Fig 3B). Furthermore, Elongin B/C, two known components of CRL5 ubiquitin E3 ligase complex, were also identified in the elutes from both FLAG-Rab40c and FLAG-Rab40c-4A, consistent with previous work that Elongin B/C binding to Rab40c is independent of LPLP motif and Cul5 (Fig 3B and E). All other putative Rab40c-binding proteins identified by mass spectrometry analysis were then filtered to eliminate possible contaminants. Only candidates that were absent in IgG control, but present in both FLAG-Rab40c and FLAG-Rab40c-4A, were identified as putative Rab40c interactors. Furthermore, all RNA, DNA, and mitochondria-binding proteins were eliminated as putative contaminants (Table S1).

Table S1. List of all proteins identified during proteomic analysis of FLAG-Rab40c immunoprecipitate.

Gene Ontology enrichment analysis of putative Rab40c interactors reveals strong enrichment for protein serine/threonine phosphatases and serine/threonine kinases, among the putative Rab40c-binding proteins (Fig 3C), suggesting that Rab40c may regulate FA-dependent signaling. Specifically, ankyrin repeat domain 28 (ANKRD28), a subunit of protein phosphatase 6 (PP6), was one of the highly enriched proteins. Importantly, other components of PP6, including ANKRD52, catalytic PP6 subunit (PP6c), and PP6R1/2, were all identified in the Rab40c precipitates (Fig 3D), indicating that the PP6 complex may interact and be regulated by Rab40c. Other PPs such as PP1CB, PP2CA, and PPM1G were also identified as putative Rab40c-binding proteins (Fig 3D); however, in the rest of this study, we will focus on interaction between PP6 complex and Rab40c.

### Rab40 interacts with the PP6 complex that contains ANKRD28 and PP6R1 subunits

**Figure 4.**
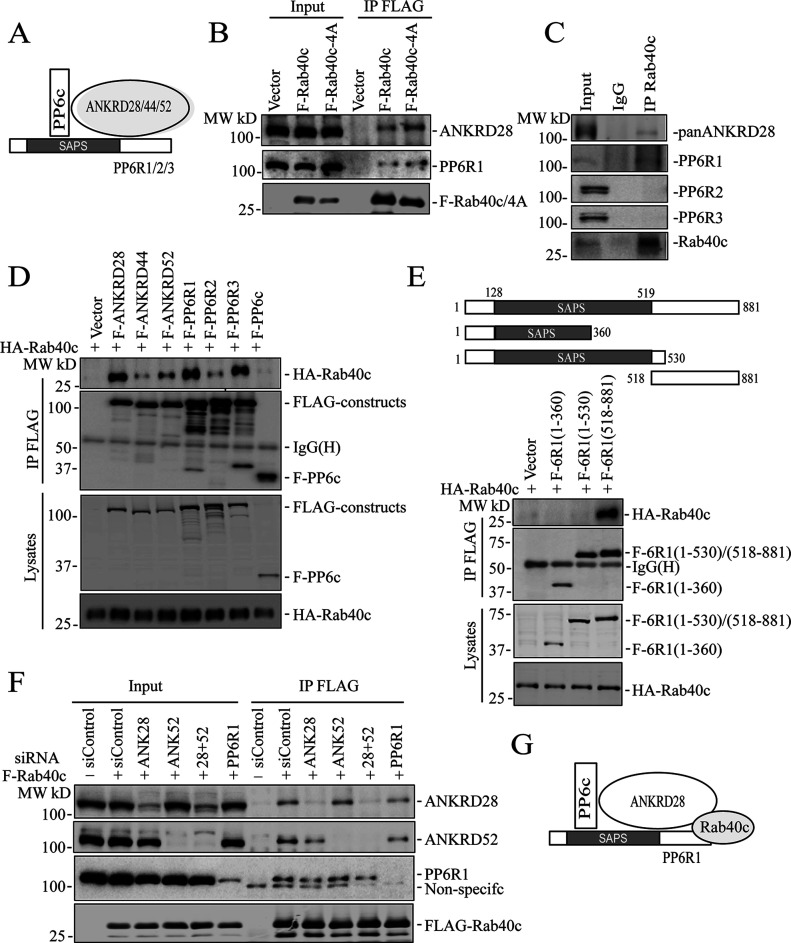
Rab40c interacts with the PP6 complex. **(A)** A model showing the PP6 complex. **(B)** MDA-MB-231 cells were transfected with control (empty plasmid), FLAG-Rab40c, or FLAG-Rab40c-4A plasmids and then cell lysates were immunoprecipitated with an anti-FLAG antibody. Cell lysates and immunoprecipitates were then immunoblotted with anti-ANKRD28, anti-PP6R1, or anti-FLAG antibodies. **(C)** MDA-MB-231 cell lysates were immunoprecipitated with anti-Rab40c and immunoprecipitates were then blotted with anti-Rab40c, anti-panANKRD, anti-PP6R1, anti-PP6R2, or anti-PP6R3 antibodies. **(D)** 293T cells were co-transfected with HA-Rab40c and control (empty plasmid) or one of FLAG-tagged PP6 components. Cell lysates were then immunoprecipitated with an anti-FLAG antibody. Cell lysates and precipitates were immunoblotted with anti-HA or anti-FLAG antibodies. **(E)** Top panels: a schematic diagram of PP6R1 deletion mutants. Lower panels: 293T cells were co-transfected with HA-Rab40c and control or one of FLAG-tagged PP6R1 deletion mutants. Cell lysates were then immunoprecipitated with an anti-FLAG antibody. Cell lysates and precipitates were immunoblotted with anti-HA or anti-FLAG antibodies. **(F)** 293T cells were co-transfected with indicated siRNA(s) and FLAG-Rab40c. Cell lysates were then immunoprecipitated with an anti-FLAG antibody. Cell lysates and immunoprecipitates were immunoblotted with indicated antibodies. **(G)** A model showing proposing that Rab40c interacts with PP6R1 and ANKRD28 subunits of PP6 complex.

The PP6 holoenzyme is a hetero-trimeric complex formed by the catalytic subunit PP6c, one of the regulatory subunits of PP6R1, 2, or 3, and one of an ankyrin repeat-domain containing protein ANKRD28, 44, or 52 (Fig 4A). PP6Rs and ANKRDs are generally considered to be regulatory and scaffolding subunits that determine the localization and specificity of PP6 complexes (Nilsson, 2019; Ohama, 2019). Thus, it is now widely accepted that there are several PP6 complexes, and that the composition of these complexes is what defines PP6 function and specificity for substrate proteins. To confirm that Rab40c binds to the PP6 complex, we overexpressed FLAG-Rab40c and FLAG-Rab40c-4A, followed by precipitation with anti-FLAG antibodies, and immunoblotting for endogenous ANKRD28 and PP6R1-3, two PP6 regulatory/scaffolding subunits that were most abundant in FLAG-Rab40c proteomic analysis (Fig 4B). Consistent with our proteomics data, we found that ANKRD28 and PP6R1 both interacted with FLAG-Rab40c and FLAG-Rab40c-4A (Fig 4B). To further confirm Rab40c and PP6 complex interaction, we immunoprecipitated endogenous Rab40c from MDA-MB-231 cells and found that ANKRDs and PP6R1, but not PP6R2 and PP6R3, were pulled out by an anti-Rab40c antibody (Fig 4C and S2A). Finally, because Rab GTPases often interact with other proteins in GTP-dependent fashion, we tested the nucleotide dependency of Rab40c and ANKRD28 interaction. To that end, we have immunoprecipitated FLAG-Rab40c after nucleotide loading of the lysates with either GDP or GTPγS (non-hydrolyzable GTP analog). As shown in Fig S2D, GTPγS enhanced ANKRD28 co-precipitation with FLAG-Rab40c suggesting that ANKRD28 may be a canonical Rab40c effector protein.

To identify which PP6 subunit mediates interaction with Rab40c, we overexpressed HA-Rab40c with all seven FLAG-tagged PP6 subunits and performed individual immunoprecipitations using an anti-FLAG antibody. Although we precipitated comparable amounts of various PP6 subunits, and HA-Rab40c was detected in all of immunoprecipitations to varying degrees, the highest amounts of HA-Rab40c co-precipitated with ANKRD28, PP6R1, and PP6R3 (Fig 4D). Interestingly, PP6R3 was also identified in Rab40c proteomic analysis (Table S1) but was eliminated from further analysis because some of the PP6R3 was also detected in IgG control. Taken together, these data suggest that Rab40c preferentially interacts with PP6 complexes containing PP6R1/ANKRD28 subunits.

It was proposed that PP6R1 functions as a scaffolding protein for PP6 holoenzyme assembly (Stefansson et al, 2008; Guergnon et al, 2009); therefore, we set out to determine which region of PP6R1 binds to Rab40c. We then generated a series of FLAG-tagged PP6R1 deletion mutants including PP6R1(1-360), PP6R1(1-530), and PP6R1(518-881) (Fig 4E), and then individually co-transfected all these constructs with HA-Rab40c, followed by co-IP with anti-FLAG and blotting with anti-HA antibodies. The results suggest that the C-terminal region of PP6R1 spanning amino acids 518–881 mediates its interaction with Rab40c (Fig 4E). Importantly, PP6R1(518-881) is outside of the SAPS domain that mediates PP6c binding to the PP6R1, suggesting that Rab40c may be able to interact with the entire PP6 holoenzyme rather than just PP6R1.

PP6 complex contains both PP6R1 and ANKRD28 subunits, and any one of them can recruit Rab40c to the PP6c/ANRD28/PP6R1 complex (Fig 4A). To identify which subunits are required for PP6 complex binding to Rab40c, we transfected 293T cells with FLAG-Rab40c and scrambled siRNA, or siRNAs targeting ANKRD28, ANKRD52, and PP6R1. FLAG-Rab40c was then immunoprecipitated, and the immunoprecipitates were analyzed for the presence of ANKD28, ANKRD52, or PP6R1 by Western blotting. As shown in (Fig 4F), siRNA-mediated knockdown effectively reduced individual protein levels >80%. However, none of these knock-downs completely eliminated FLAG-Rab40c co-precipitation with PP6. To exclude the possibility that ANKRD28 and ANKRD52 can compensate for each other, we co-depleted them using individual siRNAs. However, PP6R1 can still co-precipitate with FLAG-Rab40c in these cells (Fig 4F). Interestingly, ANKRD28 knock-down did slightly decrease the levels of PP6R1 co-precipitating with Rab40c. Similarly, PP6R1 knock-down also diminished the amount of ANKRD28 and ANKRD52 co-precipitating with Rab40c (Fig 4F). Taken together, this indicates that Rab40c likely interacts with both PP6R1 and ANKRD28 (and possibly ANKRD52). Collectively, we proposed that Rab40c interacts with the PP6R1/ANKRD28/PP6c complex by binding to the c-terminus of PP6R1, as well as ANKRD28 (Fig 4G).

### Rab40 ubiquitylates and degrades ANKRD28

**Figure 5.**
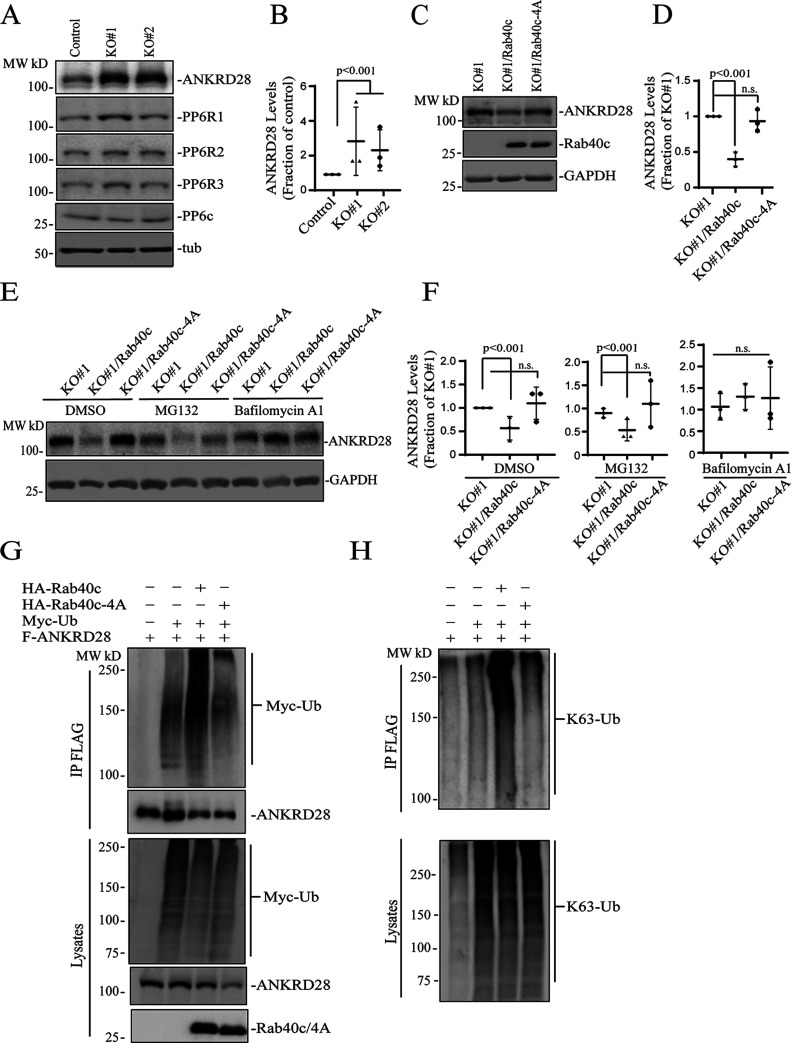
Rab40c regulates ANKRD28 ubiquitination and degradation. **(A, B)** Western blotting analysis of cell lysates from control and Rab40c-KO cells using indicated antibodies. Panel (B) shows quantification of ANKRD28 protein levels. The value shown represents the means and SEM derived from three different experiments and normalized against tubulin levels. **(C, D)** Western blotting analysis of cell lysates from Rab40c-KO, Rab40c-KO expressing FLAG-Rab40c, or FLAG-Rab40c-4A cells using indicated antibodies. Panel (D) shows quantification of ANKRD28 protein levels. The value represents the means and SEM derived from three different experiments and normalized against tubulin levels. **(E, F)** Western blotting analysis of cell lysates from Rab40c-KO, Rab40c-KO expressing FLAG-Rab40c, or FLAG-Rab40c-4A cells using anti-FLAG and anti-GAPDH antibodies. Cells were treated with DMSO, MG132 (proteosomal inhibitor), Bafilomycin A1 (lysosomal/autophagy inhibitor). **(G, H)** In vivo ANKRD28 ubiquitylation assay. 293T cells were transfected with indicated plasmids for 24 h. After treated with 100 nm Bafilomycin A1 overnight, cells were harvested and immunoprecipitated with anti-FLAG antibody followed by Western blotting for either anti-Myc, anti-FLAG, anti-HA, or anti-poly-Ub-K63 antibodies.

CRL5 complexes mediate target protein ubiquitination and subsequent degradation (Zhang & Sun, 2020; Zhao et al, 2020). Typically, the specificity of CRL5 complex is determined by the SOCS-containing subunit that serves as an adaptor between substrate proteins and CRL5. Thus, we next examined whether Rab40c/CRL5 may regulate ANKRD28 ubiquitylation and degradation. As shown in Fig 5A and B, overall cellular levels of ANKRD28 were dramatically increased in Rab40c-KO MDA-MB-231 cells, whereas having little effect on the total levels of PP6c or PP6R1-3. To further confirm the increase in ANKRD28 levels is caused by depletion of Rab40c, we generated Rab40c KO cell lines stably expressing the wild-type FLAG-Rab40c or FLAG-Rab40c-4A. As shown in Fig 5C and D, reintroduction of wild-type Rab40c into the Rab40c-KO cells decreased ANKRD28 protein level, whereas expression of FLAG-Rab40c-4A did not have any effect on ANKRD28 (Fig 5C and D), thus demonstrating that ANKRD28 protein level changes are dependent on Rab40c binding to Cul5.

**Figure S3.**
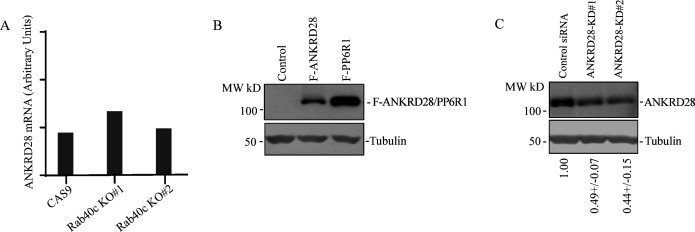
Characterization of ANKRD28 knock out. **(A)** qRT-PCR analysis of ANKRD28 mRNA levels in control and Rab40c-KO MDA-MB-231 cells. **(B)** WB analysis of lysates from MDA-MB-231 cells stably expressing FLAG-ANKRD28 or FLAG-PP6R with anti-FLAG antibody. **(C)** WB analysis of lysates from MDA-MB-231 transfected with non-targeting control siRNA or siRNA targeting ANKRD28. The numbers shown below the blot are the means and SEM derived from three independent experiments.

Next, we decided to investigate the cause for the increase in ANKRD28 levels. Consistent with the involvement of Rab40c-CRL5 in mediating the degradation of ANKRD28, mRNA levels of ANKRD28 in control and Rab40c KO cells, quantified by real-time quantitative PCR (qRT-PCR), were comparable (Fig S3A). It is well-established that ubiquitylation targets proteins for degradation in both proteasomes (K48-Ub linkage) and lysosomes (K63-Ub linkage) (Corn & Vucic, 2014; Damgaard, 2021). Thus, we next set out to determine whether Rab40c targets ANKRD28 for proteasomal or lysosomal degradation. To that end, we treated Rab40c KO cells with either lysosomal inhibitor bafilomycin A1 or the proteasomal inhibitor MG132. Surprisingly, bafilomycin A1, but not the MG132 treatment, rescued Rab40c-induced increase in ANKRD28 protein level (Fig 5E), suggesting that Rab40c/CRL5 mediates lysosomal degradation of ANKRD28, although it remains unclear whether this lysosomal targeting is mediated via autophagy or ESCRT-dependent sorting to multivesicular bodies.

Next, we asked whether ANKRD28 can be directly ubiquitylated by Rab40c/CRL5. To examine this, we first transfected 293T cells with FLAG-ANKRD28, Myc-Ub, HA-Rab40c, and HA-Rab40c-4A individually or in combinations (Fig 5G). Lysates were then immunoprecipitated with anti-FLAG antibodies and blotted for Myc-Ub with anti-Myc antibodies. When Myc-Ub was co-transfected with FLAG-ANKRD28 in the presence of the lysosomal inhibitor bafilomycin A1, the high molecular weight species were detected (presumably ubiquitylated ANKRD28), which were significantly enhanced by co-transfecting HA-Rab40c. Importantly, co-transfection of HA-Rab40c-4A with FLAG-ANKRD28 did not increase ANKRD28 polyubiquitylation, supporting that Rab40c mediates ANKRD28 ubiquitylation in a Cul5-dependent manner (Fig 5G).

Our data so far suggest that Rab40c/CRL5-dependent polyubiquitylation may target ANKRD28 to lysosomes for degradation, which is usually mediated by K63-linked polyubiquitylation. Consistent with this, Western blot analysis with anti-K63–specific anti-ubiquitin antibodies showed that Rab40c/CRL5 increases K63-linked polyubiquitylation of ANKRD28 (Fig 5H). Taken together, these results support our hypothesis that Rab40c forms a Cul5-based-ubiquitin E3 ligase complex to ubiquitylate ANKRD28 and promote its lysosomal degradation.

### ANKRD28 regulates FA formation

**Figure 6.**
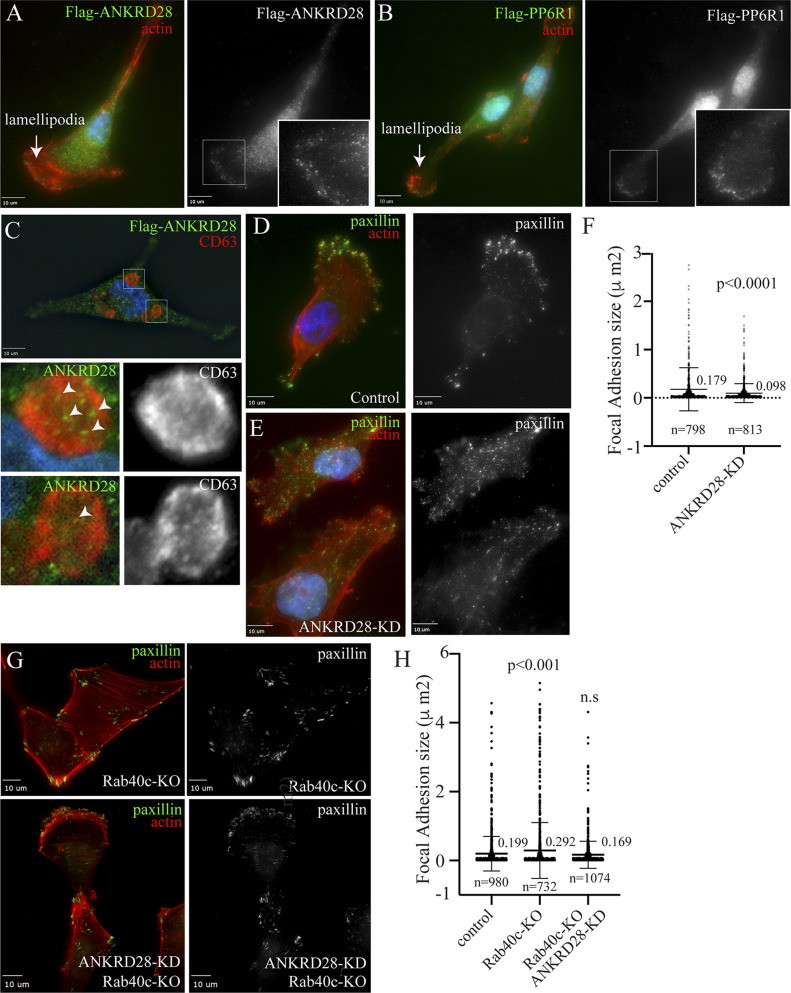
ANKRD28 regulates FA formation. **(A, B)** MDA-MB-231 cells stably expressing FLAG-ANKRD28 or FLAG-PP6R1 were plated on collagen-coated coverslips and then fixed and stained with anti-FLAG antibodies (green) and phalloidin-Alexa Fluor 594 (red). Inset regions of interest highlight ANKRD28 or PP6R1 positive puncta on the leading edge. Arrows point to the leading edge of lamellipodia. **(C)** MDA-MB-231 cells stably expressing FLAG-ANKRD28 were plated on collagen-coated coverslips and incubated with 100 nM of Bafilomycin A for 16 h. Cells were then fixed and stained with anti-FLAG (green) and anti-CD63 (red) antibodies. Inset regions of interest highlight ANKRD28 positive puncta in tye lumen of CD63-positive autophagosomes/lysosomes. **(D, E, F)** MDA-MB-231 cells were transfected with control or ANKRD28 siRNA. Cells were then plated on collagen-coated coverslips, fixed, and stained with anti-paxillin antibody (green) and phalloidin-Alexa Fluor 594 (red). Panel (E) shows quantification of FA size. n = number of FAs analyzed. Shown data are the means and SDs derived from three independent experiments. **(G, H)** Control or Rab40c-KO cells were transfected with ANKRD28 siRNA and then plated on collagen-coated coverslips and stained with anti-paxillin antibody (green) and phalloidin-Alexa Fluor 594 (red). Panel (G) shows quantification of FA size in control, Rab40c-KO, and Rab40c-KO plus ANKRD28 siRNA. n = number of FAs analyzed. Shown data are the means and SDs derived from three independent experiments.

ANKRD28 is a large scaffolding protein with 26 ankyrin repeats which has been reported to regulate cell migration (Kiyokawa & Matsuda, 2009; Tachibana et al, 2009). We therefore examined whether ANKRD28 is present at the lamellipodia where it may also regulate FAs. To that end, we first generated MDA-MB-231 cell lines stably expressing FLAG-tagged ANKRD28, or its binding partner PP6R1 (Fig S3B), to examine their localization by immunofluorescence microscopy. As shown in Fig 6A, most of the ANKRD28 protein was localized in the cytosol, but a fraction of FLAG-ANKRD28 could be observed at the lamellipodia, where it colocalizes with actin ruffles. Similarly, a sub-population of FLAG-PP6R1 could also be observed at the leading edge of the lamellipodia (Fig 6B), suggesting that PP6 complexes containing ANKRD28 and PP6R1 may function at the leading edge of the migrating cell, although additional experiments will be needed to further demonstrate that.

Our data so far suggest that Rab40c-dependent K63-ubiquitylation of ANKRD28 leads to its degradation in the lysosomes or autophagosomes. To further confirm that, we have incubated cells with bafilomycin A, a well-known inhibitor of autophagic and lysosomal degradation, to accumulate ANKRD28 in lysosomes/autophagosomes. As shown in Fig 6C, ANKRD28 puncta could be observed in an enlarged (due to bafilomycin A treatment) CD63-positive structure that likely represents either lysosomes or autophagosomes.

To test whether ANKRD28 regulates FA formation at the leading edge of the cell, we next used siRNA to knock-down ANKRD28 in MDA-MB-231 cells (Fig S3C), and then cells were stained with an anti-paxillin antibody to visualize the size and distribution of FAs. As expected, in control cells FAs were mostly present at the leading-edge of lamellipodia (Fig 6D). In contrast, in ANKRD28-depleted cells, FAs were smaller in size and situated not only at the periphery of the leading edge but scattered throughout the entire cell (Fig 6D–F). Importantly, the ANKRD28 knock-down phenotype is opposite to what was observed in Rab40c-KO cells, which generated bigger FAs. That is consistent with our hypothesis that the Rab40c/CRL5 complex regulates ANKRD28 degradation and inactivation of an ANKRD28-containing PP6 complex.

If Rab40c regulates FAs via an ANKRD28-containing PP6 complex, then ANKRD28 knockdown would be expected to at least partially reverse Rab40c-KO induced increase in FA size. To determine that, we used siRNA to knockdown ANKRD28 in Rab40c-KO cells. These cells were then fixed and stained with anti-paxillin antibodies to analyze FAs. As shown in Fig 6G and H, ANKRD28 knockdown did decrease FA size in Rab40c-KO cells. This is consistent with the hypothesis that Rab40c affects FA formation by regulating ANKRD28 degradation and activity of ANKRD28-containing PP6 complexes at the lamellipodia of migrating cells.

### Rab40c and ANKRD28 regulate FAK and hippo signaling pathways during cell migration

**Figure S4.**
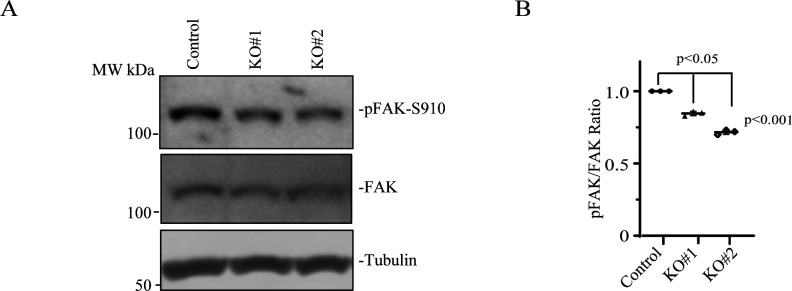
The effect of Rab40c KO on the levels of pFAK-S190. **(A)** Cell lysates from control and Rab40c-KO cells were immunoblotted with anti-pFAK-S190, anti-FAK, and anti-tubulin antibodies (A). **(B)** Panel (B) shows quantification of the levels of pFAK-S190 phosphorylation. Data shown are the means and SEM derived from three independent experiments.

Our data so far suggest that Rab40c regulates FA formation during cell migration. This regulation is presumably mediated by ANKRD28/PP6-dependent de-phosphorylation of specific target proteins that regulate FAs. Thus, next we set out to identify the identity of the target proteins that are directly or indirectly regulated by the ANKRD28/PP6 complex. One of the well-established key regulators of FA dynamics is FAK (Katoh, 2020; Martínez et al, 2020). Importantly, FAK is tightly regulated by several tyrosine and serine/threonine kinases, thus, could be a candidate for ANKRD28/PP6-dependent de-phosphorylation. Among several Ser/Thr phosphorylation sites, Ser910 has emerged as one of the key regulators of FAs (Villa-Moruzzi, 2007; Zheng et al, 2009). Specifically, phosphorylation of FAK-Ser910 was shown to increase FA turnover, presumably by regulating recruitment of paxillin and several tyrosine phosphatases that then de-phosphorylate Y392. To test whether Rab40c may regulate FAs by inhibiting ANKRD28/PP6-dependent Ser910 de-phosphorylation, we have compared the levels of pFAK-Ser910 in control and Rab40c-KO cells. As shown in Fig S4, Rab40c depletion did decrease the levels of pFAK-Ser910. That, at least in part, would contribute to the increase in FA size and number in Rab40c-KO cells, although further studies will be needed to determine whether ANKRD28/PP6 directly de-phosphorylates pFAK-Ser910.

**Figure 7.**
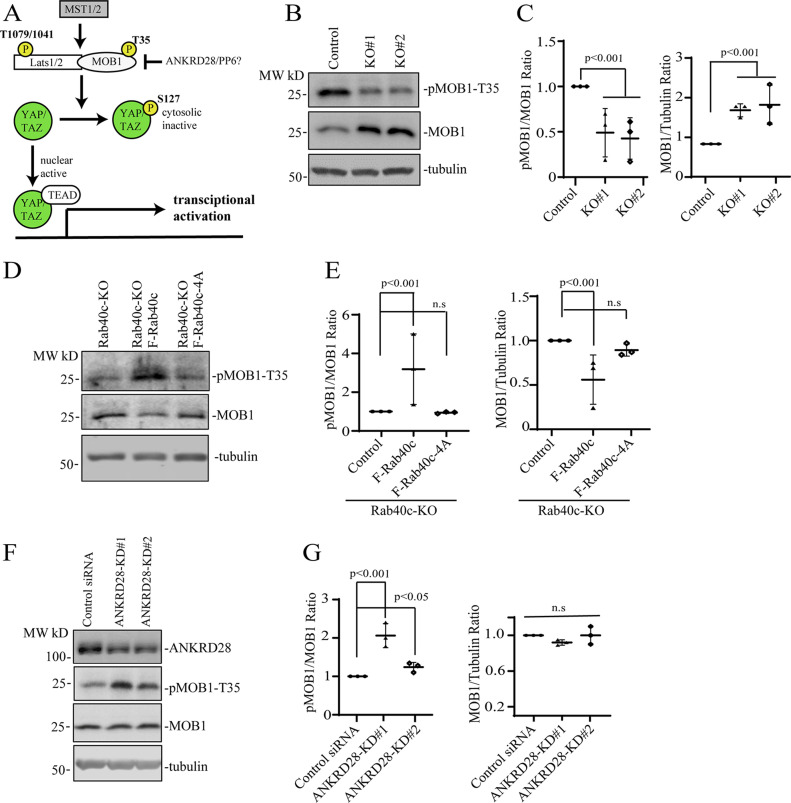
Rab40c and ANKRD28 regulates MOB1 phosphorylation. **(A)** A model showing Hippo-signaling pathway and a potential target of ANKRD28/PP6 complex. **(B, C)** Immunoblotting of cell lysates from control and Rab40c-KO cells with anti-pMOB1-T89, anti-MOB1, and anti-tubulin antibodies. Panel (C) shows quantification of pMOB1/MOB1 and MOB1/tubulin ratio. The data shown represent the means and SEM derived from three different experiments and normalized against tubulin levels. **(D, E)** Immunoblotting of cell lysates from Rab40c-KO, Rab40c-KO–expressing FLAG-Rb40c, or FLAG-Rab40c-4A cells with anti-pMOB1-T35, anti-MOB1, and anti-tubulin antibodies. Panel (E) shows quantification of pMOB1/MOB1 and MOB1/tubulin ratio in (D). The data shown represent the means and SEM derived from three different experiments and normalized against tubulin levels. **(F, G)** MDA-MB-231 cells were transfected with ANKRD28 siRNA or non-targeting control. Cell lysates were then immunoblotted with indicated antibodies. Panel (G) shows quantification of pMOB1/MOB1 and MOB1/tubulin ratio. The shown data represent the means and SEM derived from three different experiments and normalized against tubulin levels.

Recently, the Hippo-signaling pathway was reported to play an important role in mechanosensing ECM stiffness and regulating FAs dynamics (Nardone et al, 2017; Mason et al, 2019; Wang et al, 2021). Importantly, ANKRD28 has been suggested to bind MOB1, the key regulator of the Hippo pathway (Fig 7A) (Couzens et al, 2013; Rusin et al, 2015; Ohama, 2019). However, whether ANKRD28 actually regulates Hippo-signaling has not been explored, and our data raise a very interesting possibility that Rab40c regulates Hippo signaling at the FAs through inactivation of ANKRD28/PP6 complex. MOB1 functions as an activator of Lats1 (Fig 7A), and MOB1 phosphorylation by MST1/2 increases its ability to activate Lats1 (Ni et al, 2015; Gundogdu & Hergovich, 2019; Delgado et al, 2020; Duhart & Raftery, 2020). Consistent with the possibility that Rab40c may regulate the Hippo pathway, we found that phosphorylation of MOB1 at Thr35 was significantly decreased, whereas the total protein level significantly increased in Rab40c-KO cells (Fig 7B and C). Furthermore, reintroduction of wild-type Rab40c, but not Rab40c-4A mutant, into the Rab40c-KO cells rescued MOB1 phosphorylation defects and decreased the total protein level of MOB1, suggesting that changes in MOB1 phosphorylation and total protein levels may be mediated by Rab40c/CRL5 complexes (Fig 7D and E). To further confirm that changes in MOB1 phosphorylation in Rab40c-KO cells are mediated by ANKRD28, we used two different siRNAs to knock-down ANKRD28. As shown in Fig 7F and G, in control cells knock-down of ANKRD28 increased the levels of pMOB1-T35, again supporting the hypothesis that the ANKRD28/PP6 complex regulates MOB1-T35 phosphorylation. Collectively, these data demonstrate that Rab40c may regulate MOB1 phosphorylation through regulating ANKRD28 ubiquitylation and degradation, thus inhibiting ANKRD28/PP6 complex activity.

**Figure 8.**
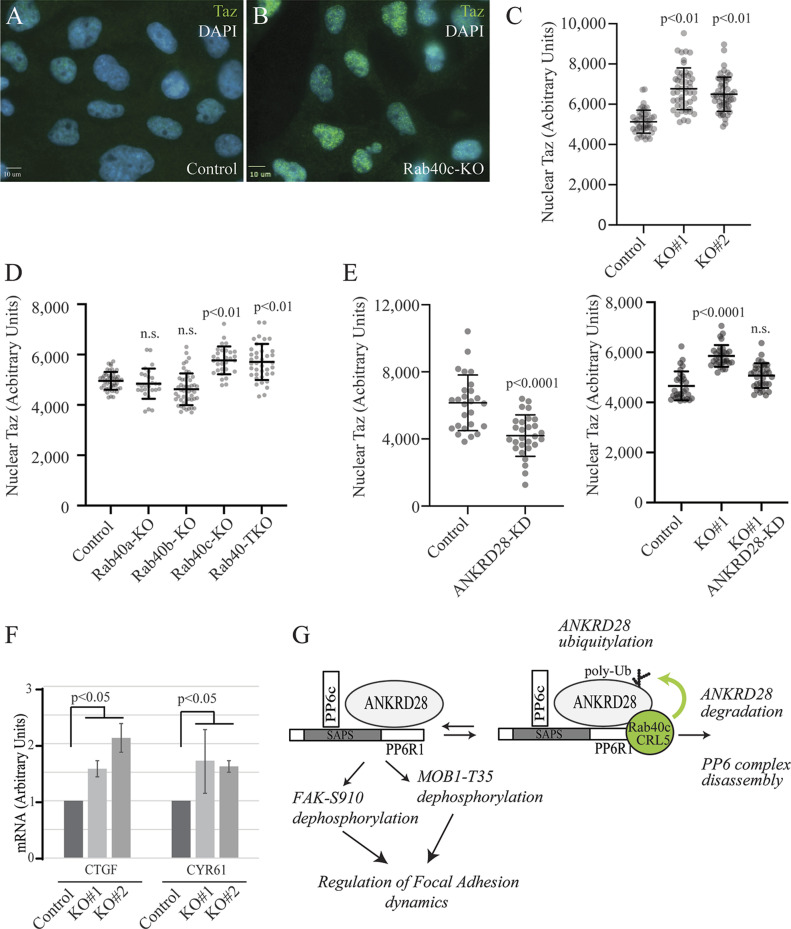
Rab40c regulates Hippo signaling in MDA-MB-231 cells. **(A, B, C)** Control and Rab40c-KO MDA-MB-231 cells were plated on collagen-coated coverslips. Cells were then fixed and stained with anti-TAZ antibody (green) and DAPI (blue). Panel (C) shows quantification of nuclear TAZ fluorescence intensity. Data shown are the means and SDs derived from three independent experiments. **(D)** Quantification of nuclear TAZ fluorescence intensity in control, Rab40c-KO, Rab40b-KO, Rab40c-KO, and Rab40-TKO cells stained as described in (A). **(E)** Quantification of nuclear TAZ fluorescence intensity in MDA-MB-231 cells transfected with ANKRD28 siRNA or non-targeting control (left panels). Quantification of nuclear TAZ fluorescence intensity in control or Rab40c-KO cells transfected with either non-targeting control siRNA or Rab40c ANKRD28 siRNA (right panels). **(F)** qRT-PCR analysis of CTGF and CYR61 mRNAs (known Hippo pathway targets) in control and Rab40c-KO MDA-MB-231 cells. **(G)** Proposed model for Rab40c function in regulation FAs dynamics. The PP6 complex containing ANKRD28 and PP6R1 dephosphorylates FAK-S910 and/or pMOB1-T35 to regulate FAs dynamics. When Rab40c binds to PP6 through ANKRD28 and PP6R1, it leads to ANKRD28 ubiquitylation and subsequent degradation which results in PP6 disassembly and inactivation.

Our findings that Rab40c regulates MOB1 protein levels and phosphorylation prompted us to test whether Hippo downstream transcription factors YAP and TAZ are also activated, because LATS1/MOB1 complex mediates YAP/TAZ phosphorylation (Meng et al, 2016; Battilana et al, 2021). Phosphorylation of YAP/TAZ acts as a negative regulator of YAP/TAZ by keeping them in cytosol and away from the nucleus (Rausch & Hansen, 2020; Kwon et al, 2021). Because of a significant similarity between YAP and TAZ in their sequences, it is challenging to find highly specific YAP antibodies, thus we focused on TAZ. Given that nuclear-cytoplasmic localization reflects TAZ activity, we used immunofluorescence staining to examine the localization of TAZ. As shown in Fig 8A–C, Rab40c-KO increased TAZ translocation from the cytoplasm into the nucleus. The increase seems to be Rab40c-KO specific because neither Rab40a nor Rab40b knock-out led to a similar increase in nuclear TAZ (Fig 8D) and knocking out all three Rab40 isoforms (TKO) did not further increase the nuclear TAZ as compared with Rab40c-KO cells (Fig 8D).

Because Rab40c/CRL5 mediates ubiquitylation-dependent degradation of ANKRD28, we next examined whether ANKRD28 is a positive TAZ regulator, and whether ANKRD28 knockdown inhibits TAZ nuclear localization. As shown in Fig 8E, knockdown of ANKRD28 by siRNA significantly reduced nuclear TAZ in MDA-MB-231 cells. Importantly, knockdown of ANKRD28 in Rab40c-KO cells reversed Rab40c-KO–induced activation of TAZ (Fig 8E). Taken together, our data are consistent with the hypothesis that Rab40c-KO promotes TAZ nuclear localization by increasing ANKRD28/PP6–dependent de-phosphorylation of Hippo signaling pathways regulators, such as MOB1.

Nuclear TAZ interacts with transcription factor TEAD to operate as a coactivator to increase transcription of their many target genes, including CTGF and CYR61. If Rab40c-KO leads to an increase in nuclear TAZ, one would predict that Rab40c-KO should also increase the transcription of TAZ target genes. Consistent with this, we found the mRNA expression levels of CTGF and CYR61 were higher in Rab40c-KO than in the control cells, as determined by qRT-PCR analysis (Fig 8F). Altogether, our data support that Rab40c forms a Cul5-based ubiquitin E3 ligase to ubiquitylate and degrade ANKRD28. This then results in inhibiting ANKRD28/PP6 complex activity and subsequent increases in phosphorylation of several important signaling molecules including FAK and MOB1, thus regulating FAs dynamics during cell migration.

## Discussion

Rab40c belongs to a unique Rab40 subfamily of small monomeric GTPases that contain SOCS domain at their C-terminus, thus binding Cul5 to form a ubiquitin E3 ligase complex (Rab40/CRL5) to control protein ubiquitylation and degradation (Duncan et al, 2021). All vertebrates express two Rab40 family isoforms, Rab40b and Rab40c. Although Rab40b and Rab40c are closely related isoforms, it has been suggested that they may ubiquitylate and regulate different, but probably overlapping, subsets of target proteins. Indeed, RACK1 and Varp, the only two proposed Rab40c/CRL5 target proteins, do not appear to be ubiquitylated by Rab40b (Yatsu et al, 2015; Day et al, 2018; Linklater et al, 2021), whereas Rap2 GTPase appears to be ubiquitylated predominately by Rab40b. Thus, determining the repertory of Rab40b and Rab40c-specific target proteins will be the key to understanding functions of the Rab40 subfamily of small monomeric GTPases and is one of the goals of this study.

Regulating cell migration recently emerged as one of the major functions of the Rab40 subfamily of GTPases. It has been shown that Rab40b/CRL5 regulates cell motility and invasion by ubiquitylation of EPLIN (Linklater et al, 2021), as well as by mediating MMP2 and MMP9 transport to the invadopodia (Jacob et al, 2013). Similarly, Rab40a, an isoform expressed only in higher primates, regulates degradation of RhoU (Dart et al, 2015), one of the regulators of cell adhesion to ECM. However, it remains unclear whether Rab40c is also involved in regulating cell motility, and what proteins could be its targets of ubiquitylation. In this study, we show that Rab40c plays an important role in controlling FA formation during cell migration because Rab40c depletion significantly increases the number and size of FAs, while also disrupting polarization of FA formation at the leading edge of lamellipodia. Furthermore, although Rab40c was previously suggested to regulate lipid droplet formation (Tan et al, 2013; Luo et al, 2017), we did not find any evidence that Rab40c plays a similar role in migrating MDA-MB-231 cells. Thus, taken together, all these data suggest that all Rab40 subfamily isoforms regulate, at least in part, actin and FA dynamics during cell migration by ubiquitylating and regulating isoform-specific target proteins.

Rab proteins are key regulators of intracellular membrane trafficking, and each Rab protein has a distinct location corresponding to its functions. Here, we show that Rab40c localizes at two specific compartments: Golgi, and the leading edge of the lamellipodia, implying that Rab40c may regulate membrane trafficking from the Golgi to the cell surface, as well as plasma membrane and actin dynamics during migration. In line with this, we found that Rab40c-KO cells form more and bigger FAs, which evenly distribute throughout the cell. FAs are integrin-containing, multi-protein structures that link the intracellular cytoskeleton to the ECM. The number and localization of FAs are tightly controlled during cell migration, with coordinated assembly and turnover of FAs at the front of the migrating cell body and disassembly at the rear. Intriguingly, Rab18, an ER-resident protein, regulates kinectin-1 transport toward the cell surface to form ER–FA contacts, thus promoting FA growth during cell migration. Rab18 is closely related to the Rab40 family, and it has been suggested that Rab40 family proteins are expanded from ancestral Rab18 during metazoan evolution. Thus, it is not surprising that they share a partially redundant role in some cellular processes.

The Rab40 subfamily of GTPases have a conserved SOCS domain at their C-terminus, and it has been shown that Rab40b binds directly to Cul5 and its accessory proteins Elongin B/C (Linklater et al, 2021; Duncan et al, 2022). Here we show that human Rab40c also binds Cul5 and Elongin B/C and that this binding is blocked by mutating a Cul-box within the SOCS domain. We also identified several putative Rab40c-interacting proteins, including protein phosphatase 6 (PP6) complex. PP6 is heterotrimeric complex that belongs to the serine/threonine phosphatase family, comprising a single catalytic subunit (PP6c), one of a PP6 regulatory subunit (PP6R1, 2, or 3), and one of an ankyrin repeat domain scaffolding subunit (ANKRD28, 44, or 52) (Stefansson et al, 2008; Ohama, 2019). PP6 complex plays an important role in many fundamental cellular processes, but the regulatory mechanism for PP6 complex activity remains largely unknown. Because the catalytic PP6 subunit appears to be quite promiscuous, it has been proposed that the specificity of PP6 activity is regulated by its scaffolding (ANKRD28, 44, and 52) and regulatory (PP6R1-3) subunits. Thus, it has been hypothesized that specific ANKRD and PP6R subunits define spatiotemporal properties of PP6 complex activity and determine the target protein specificity. However, so far, we know very little about specific functions of different ANKRD and PP6R subunits. In this study, we found that Rab40c interacts with the PP6 complex, which leads to ubiquitylation and lysosomal/autophagic degradation of its scaffolding subunit ANKRD28. Although which subunit is responsible for directly recruiting Rab40c to the PP6 complex needs to be further assessed, our data suggest that Rab40c co-binds to both ANKRD28 and PP6R1, likely recruiting Rab40c to the assembled PP6 complex (Fig 8G). Importantly, regulation of ANKRD28 by Rab40c is Cul5 dependent because Rab40c, but not Rab40c-4A mutants, can enhance ANKRD28 ubiquitylation.

Because, in addition to the plasma membrane, Rab40c also localizes to the Golgi, we examined the subcellular localization of ANKRD28 and PP6R1. However, we did not observe ANKRD28 or PP6R1 at the Golgi, but instead we found that both these proteins are present at the leading edge of lamellipodia. Consequently, we hypothesize that Rab40c/CRL5 may regulate FAs through induction of localized ANKRD28 degradation that would lead to localized disassembly and inactivation of ANKRD28 and PP6R1-containing PP6 complexes at the leading edge of lamellipodia. In fact, ANKRD28 has been previously implicated in regulation of FA dynamics and cell migration (Kiyokawa & Matsuda, 2009; Tachibana et al, 2009), although how ANKRD28 affects FA dynamics remains largely unclear. In this study we found that ANKRD28 knockdown leads to a decrease in FA size and a loss of polarized FA distribution at the leading edge of the migrating cell. Importantly, depletion of ANKRD28 in Rab40c KO cells partially restored both the size and distribution of FAs, suggesting that the Rab40c-KO phenotype was, at least partially, due to an increase in ANKRD28 protein level and presumably over-activation of PP6 complex at the leading edge.

ANKRD28 is an essential scaffolding component of the PP6 complex, thus we predicted that Rab40c-dependent ubiquitylation and degradation of ANKRD28 leads to localized inactivation of a specific subset of PP6 (PP6R1/ANKRD28/PP6c), in turn controlling the phosphorylation of some signaling molecules important for FAs dynamics. It is well established that FAK-Src signaling plays a crucial role in regulating FAs dynamics. Phosphorylation of FAK-S910, which is mediated by ERK, promotes FAK dephosphorylation at Y397, and is necessary for invasive cell migration (Zheng et al, 2009). We found pFAK-S910 levels are decreased in Rab40c-KO cells, whereas total FAK remained constant. That raises an intriguing possibility that ANKRD28/PP6 directly dephosphorylates pFAK-S910. Another possibility is that ERK activity is decreased in Rab40c-KO cells because PP6 has recently been identified as a key negative regulator of ERK signaling by dephosphorylating MEK (Cho et al, 2021). Thus, although additional studies will be needed to determine the mechanisms of PP6-dependent FAK regulation, our data imply that the Rab40c-ANKRD28/PP6 pathway contributes to regulating FAK activity and FA disassembly during cancer cell migration (Fig 8G).

PP6 complex was also identified in interactome analysis of the Hippo signaling pathway, and it was proposed that PP6 may be a regulator of Hippo-signaling (Couzens et al, 2013; Ohama, 2019; Emami et al, 2020). However, the precise role of PP6 in the Hippo pathway remains unknown. Importantly, interactions between the Hippo signaling pathway components YAP/TAZ and FAs have been revealed recently (Nardone et al, 2017; Mason et al, 2019; Wang et al, 2021). It was shown that FAs act as a hub for sensing and transmission of mechanical cues to regulate YAP/TAZ activation. In turn, YAP/TAZ regulates cell mechanics by controlling FA assembly through co-transcription of genes encoding for various FA regulators. Based on these data, we examined the possibility that Rab40c-ANKRD28/PP6 directly regulates Hippo-signaling to control FAs dynamics. Consistent with this hypothesis, pMOB1-T35 levels decreased in Rab40c-KO cells. In contrast, knocking down ANKRD28 increased pMOB1-T35. Finally, Rab40c-KO–induced decrease in MOB1 phosphorylation could be rescued by an overexpression of wild type, but not Cul5-binding mutants, of Rab40c.

MOB1 is a key regulator of large tumor suppressor 1/2 (Lats1/2) kinases, and phosphorylation of pMOB1-T35 promotes its binding to Lats1/2 (Couzens et al, 2013; Meng et al, 2016). Phosphorylation of YAP and TAZ by Lats/MOB1 kinase complex results in YAP/TAZ cytoplasmic retention and inhibits their transcriptional activities. Consistent with this, we confirmed that Hippo-signaling is affected in Rab40c-KO cells by TAZ immunostaining and qRT-PCR quantification of YAP/TAZ target genes. More interestingly, a recent study showed that Rab40c is down-regulated upon YAP stimulation (Moon et al, 2020), thus the Rab40c-PP6 and YAP/TAZ may constitute a feed-forward loop to regulate FAs dynamics in migrating cells. Taken together, our data suggest that the Cul5/Rab40c-ANKRD28/PP6 axis is an important regulator of Hippo-signaling and FAs dynamics (Fig 8G).

Although our data demonstrate that Rab40c-ANKRD28/PP6 affects FAs formation by co-regulating FAK and Hippo-YAP/TAZ signaling (Fig 8G), many questions remain to be addressed in the future. For example, it remains completely unknown what regulates formation and activity of the Rab40c/CRL5 complex, and whether this complex has distinct functions at the plasma membrane and Golgi. Can Rab40c-PP6 regulate vesicles (like MMP2/9-containing secretory vesicle) trafficking? In addition, it is becoming clear that Rab40/CRL5 complexes regulate ubiquitylation of multiple proteins, thus what are other Rab40c/CRL5 targets and what are their functions? Finally, do our findings have any clinical implications? Given about 10% of melanoma patients harbor PP6c inactivating mutations, and dysregulated Hippo pathways are also associated with various diseases, especially with cancer, answering these questions will be very interesting and be the focus of future studies.

## Materials and Methods

### Cell culture and transfection

All cell lines were cultured as described previously (Jacob et al, 2013). Briefly, human embryonic kidney 293T cells were grown in complete DMEM (supplemented with 10% fetal bovine serum and 100 μg/ml of penicillin and streptomycin) at 37°C in a 5% CO_2_ atmosphere. MDA-MB-231 cells were grown in complete DMEM supplemented with 1 μg/ml human recombinant insulin, 1% non-essential amino acids, and 1% sodium pyruvate. Cell lines were routinely tested for mycoplasma. All cell lines used in this study were authenticated and are in accordance with American Type Culture Collection standards. 293T cells were grown to 70–80% confluence and transfected using the Lipofectamine 2000 (Invitrogen) transfection reagent according to the manufacturer’s instructions. MDA-MB-231 cells were grown to 80–90% confluence and transfected using JetPRIME (Polyplus). Lipofectamine RNAiMAX (Invitrogen) was used for transfection of siRNAs both in 293T and MDA-MB-231 cells.

### Mammalian expression constructs

Human Rab40a, Rab40-b, and Rb40c plasmids were purchased from the Functional Genomics Core Facility at the University of Colorado. Human ANKRD28/44/52, PP6R1/2/3, PP6c, and Myc-Ub were described previously (Han et al, 2012; Couzens et al, 2013). Expression plasmids of GFP-Rab40c, FLAG-Rab40c, HA-Rab40c, and FLAG-PP6R1 deletion mutants were constructed by PCR, followed by subcloning into the pRK7 or pGPS vector containing an N-terminal FLAG or HA tag. FLAG-Rab40c-4A mutant was generated by PCR using the following primers (Integrated DNA Technologies): Forward: GTCGTCGACATGGGCTCGCAGGGCAGTCCGGTG, Reverse: TGCAGCCTTGTCGATGAGG and Forward: GCGGCCGTCACCATCAAG, Reverse: TAGCGGCCGCTAGGAGATCTTGCAGTTAC. All plasmids were validated by DNA sequencing.

### Antibodies

The following antibodies were used in this study: anti-FLAG (clone M2, WB 1:1,000; Sigma-Aldrich), anti-GAPDH (WB 1:5,000; UBPBio), FAK S910 (WB 1:1,000, 44-596G; Invitrogen), total FAK (WB 1:1,000, 610087; BD Biosciences), and paxillin (IF 1:500; Transduction Labs). Anti-HA (WB 1:500, IP 2 μg/1 mg cell lysate, SC F-7), anti–α-tubulin (WB 1:5,000, 23948), anti-Rab40c (WB 1:500, H-8 sc514826), cul-5 (WB 1:500, H-300), and mouse ANKRD28 (WB 1:500) were purchased from Santa Cruz Biotechnology. MOB1(E1N9D) and p-MOB1(D2F10) were purchased from Cell Signaling Technology. Rabbit anti-SAPS1/2/3, ANKRD28/52, and PP6c were purchased from Bethyl Laboratories. Specificity of anti-Rab40c antibody was confirmed by immunoblotting lysates derived from cells expressing FLAG-Rab40a, FLAG-Rab40b, or FLAG-Rab40c constructs (Fig S2A).

### Identification of Rab40c-interacting protein

A lentivirus-based method was used to generate stable cell lines expressing FLAG-Rab40c or FLAG-Rab40c-4A as described previously (Han et al, 2012). Putative Rab40c-binding proteins were identified by coimmunoprecipitation using anti-FLAG antibody–coated beads as described previously (Prekeris et al, 2000). Briefly, 50 μg affinity purified anti-FLAG antibody was bound to 100 μl Protein G–Sepharose beads. Antibodies were then cross-linked to beads using dimethyl pimelimidate dihydrochloride. Anti-FLAG antibody beads were then incubated with 2 ml of 1 mg/ml Triton X-100 cellular lysates (PBS, 1% Triton X-100, and 10 mM PMSF), followed by a wash with 5 ml PBS. Proteins were eluted from anti-FLAG antibody beads with 1% SDS and then analyzed using tandem mass spectrometry (Proteomics Core on campus). The UniProtKB/SwissProt human database was used for protein identification. Nonspecific contaminants were identified and eliminated by the following criteria: (1) presence in IgG control; (2) presence in the CRAPome database and (3) all RNA-, DNA-binding proteins, and mitochondrial proteins were considered a contaminant.

### CRISPR-Cas9 knockout lines

Guide RNAs for Rab40c targeting 59-TACCGTTACTGTAGGCGTAC-39 (exon 1) and 59-AGGTAGTCGTAGCTCTTCAC-39 (exon 3) were transfected into MDA-MB-231 cell line with Dox-induced Cas9. Cells were split 24 h after transfection and seeded for single colonies and then were screened by Western Blotting, followed by PCR cloning and genotyping (Fig S2B and C). Rab40a, b, or triple knockout lines has been described previously (Linklater et al, 2021). For each knockout line, two different clones were used for all experiments.

### GTPγS loading of cell lysates

MDA-MB-231 lysates were generated by extracting cells with PBS containing 1% Triton X-100. Lysates were then incubated with 1 mM EDTA for 1 min at 37°C to chelate all magnesium and to dissociate GDP or GTP from all small monomeric GTPases, including FLAG-Rab40c. Afterwards, lysates were incubated with 5 mM MgCl_2_ in the presence of 1 mM GDP or 1 mM GTPγS for 10 min at 37°C to re-load FLAG-Rab40c with either GDP or GTPγS.

### qRT-PCR

Total RNA was extracted using TRIzol (Invitrogen) according to the manufacturer’s protocol. Reverse transcription to cDNA was performed with SuperScript III (Invitrogen) using random hexamer primers. qRT-PCR was performed using iTaq SYBR Green qPCR Master Mix on Applied Biosystems ViiA7 Real Time PCR System. The qRT-PCR amplification conditions were 50°C (2 min), 95°C (10 min), 40 cycles at 95°C (15 s), and 60°C (1 min). Targets were normalized to GAPDH. The following primers used for qRT-PCR: ANKRD28 forward: ACTGCTCTCCACGGTAGATTC and reverse: GGGGAACATTCCATGTATGCC; CTGF forward: ACCGACTGGAAGACACGTTTG and reverse: CCAGGTCAGCTTCGCAAGG; CYR61 forward: AGCCTCGCATCCTATACAACC and reverse: TTCTTTCACAAGGCGGCACTC; GAPDH forward: CTGGGCTACACTGAGCACC and reverse: AAGTGGTCGTTGAGGGCAATG.

### Immunoprecipitation and immunoblotting assays

For non-denaturing immunoprecipitation, cells in a 100-mm dish were harvested and washed with 1× PBS, then lysed with 1.0 ml ice-cold cell lysis buffer (20 mM Tris–HCl, pH 7.6, 150 mM NaCl, 2 mM EDTA, 1% Triton X-100, and 10% glycerol) with protease inhibitor cocktails (Roche). After clearing lysates by centrifugation, supernatants were incubated with 2 μg of an appropriate antibody or control IgG for 4 h at 4°C, then supplemented with 50 μl protein G beads. After overnight rocking, protein G beads were pelleted by centrifugation and washed three times with the cell lysis buffer plus 0.5 M NaCl. Bound proteins were eluted in 50 μl 1× SDS sample buffer.

For denaturing immunoprecipitation (used in ubiquitylation assays), cells in a 100-mm dish were lysed in 1-ml cell lysis buffer plus 1% SDS. Cell lysates were collected and then heated at 95°C for 15 min. After centrifugation, supernatants were diluted with the cell lysis buffer to reduce SDS concentration to 0.2%. The immunoprecipitation assay was performed as described above, except that 5 μg anti-FLAG antibody was used in each reaction. Eluates (40 μl) were resolved in SDS–PAGE and transferred to nitrocellulose membranes for immunoblotting assays. Immunoblotting images were captured using a ChemiDoc MP Imaging system (Bio-Rad). All uncropped and unmodified Western blots are shown in Source Data for Figs 1 and 2.

### Ubiquitylation assay

Ubiquitylation assay was performed as described previously (Linklater et al, 2021). Briefly, 293T cells (∼80% confluency) were transfected with plasmids expressing pRK5-FLAG-ANKRD28 with or without pRK5-Myc-Ub, pRK7-HA-Rab40c, or pRK7-HA-Rab40c-4A using Lipofectamine 2000. After 24 h, cells were treated with 100 nM Bafilomycin-A1 (S1413; Selleckchem) overnight. Then, cells were lysed in 1% SDS for denaturing immunoprecipitation as described above. Bound proteins were eluted in 50 μl 1X SDS sample buffer. Eluates (20 μl) were resolved via SDS–PAGE and transferred to nitrocellulose membranes for immunoblotting. Blot images were captured using a ChemiDoc MP Imaging system (Bio-Rad).

### siRNA knockdown

For ANKRD28/52 and PP6R1 knockdown, siRNAs were purchased from Sigma-Aldrich. Mission siRNA universal negative control (SIC001; Sigma-Aldrich), ANKRD28 siRNAs (SASI_Hs01-00173856) and (SASI_Hs01_00173857), ANKRD52(SASI_Hs02_00368435) and PP6R1(SASI_Hs01_00222781) were transfected using Lipofectamine RNAiMAX (Invitrogen) according to the manufacturer’s protocol.

### Immunofluorescent microscopy

MDA-MB-231 cells were seeded onto collagen-coated glass coverslips and grown in full growth media unless otherwise noted for at least 24 h. Cells were washed with PBS and fixed in 4% paraformaldehyde for 15 min. Samples were then washed three times in PBS then incubated in blocking serum (1× PBS, 5% normal donkey serum, and 0.3% Triton X-100) for 1 h at room temperature. Primary antibodies were then diluted at 1:100 in dilution buffer (1× PBS, 1% BSA, and 0.3% Triton X-100) overnight at 4°C. Samples were then washed three times with PBS and incubated with fluorophore-conjugated secondary antibodies (1:100 in dilution buffer) for 1 h at room temperature. Cells were then washed three times in PBS and mounted onto glass slides. Cells were then imaged on an inverted Zeiss Axio Observer deconvolution microscope with a 63× oil immersion lens.

### Image analysis

#### Focal adhesion analysis

To analyze FA size and number, cells were fixed and co-stained with anti-paxillin antibody and phalloidin-Alexa 596. For analysis, cells were randomly selected (using phalloidin-Alexa 596 channel) in at least five image fields (typically 10–30 cells were analyzed for each experimental condition) using the following criteria: (1) cell was not contacting any of the surrounding cells; (2) cell has clearly identifiable lamellipodia. All images were then acquired using the same exposure time. To select FAs, masks were created by image fragmentation and thresholding using anti-paxillin staining channels. The same thresholding and fragmentation criteria were used in all images. The size and number of the FAs were then measured using Intelligent Imaging Innovations 3I Imaging software. Data were derived from at least three independent experiments. Around 800–1,000 FAs were analyzed for each experimental condition.

#### Cell-ECM adhesion analysis

To analyze cell-ECM, adhesion cells were plated on collagen-coated glass coverslips and incubated for 30, 60, or 90 min. Cells were the n fixed and stained with DAPI and phalloidin-Alexa 596. For analysis, 10–15 cells for each condition were randomly selected (using DAPI channel) using the following criteria: (1) cell was not contacting any of the surrounding cells. All images were then acquired using the same exposure time. To measure adhesion area, masks were created using a phalloidin-Alexa 596 staining channel. The surface area of attachment for each cell was then measured using Intelligent Imaging Innovations 3I Imaging software. Data were derived from at least three independent experiments.

#### TAZ activation analysis

To analyze nuclear TAZ localization, the cells were fixed and co-stained with anti-TAZ antibody and phalloidin-Alexa 596. For analysis, five random image fields (using phalloidin-Alexa 596 channel) were selected, and all cells were analyzed in each field (typically 20–30 were cells analyzed for each experimental condition). All images were then acquired using the same exposure time. To select the nucleus, masks were created by image fragmentation and thresholding using DAPI staining channels. The same thresholding and fragmentation criteria were used in all images. The TAZ fluorescence sum intensity in the nucleus was then measured using Intelligent Imaging Innovations 3I Imaging software and expressed as fluorescence intensity per μm^2^. Data shown were derived from at least three independent experiments.

### Statistical analysis

Statistical analysis for all experiments was determined using GraphPad Prism Software (GraphPad). Datasets were assessed for normal distribution using the Shapiro-Wilk normality test. A two-tailed *t* test was performed on all normally distributed datasets and a Mann–Whitney U test was performed for datasets not normally distributed. Data were collected from at least three independent experiments unless otherwise noted. In all cases, *P* ≤ 0.05 was regarded as significant. Error bars represent SD unless otherwise noted. For all immunofluorescence experiments, at least five randomly chosen image fields per condition were used for data collection. For quantitative immunofluorescence analysis, the same exposure was used for all images in that experiment and quantified using Intelligent Imaging Innovations 3I Imaging software.
