# Supplementary figures and images for: Rab40c regulates focal adhesions and PP6 activity by controlling ANKRD28 ubiquitylation
Source: Life Sci Alliance. 2022 May 5;5(9):e202101346. doi: 10.26508/lsa.202101346 (PMC9070665; doi:10.26508/lsa.202101346)

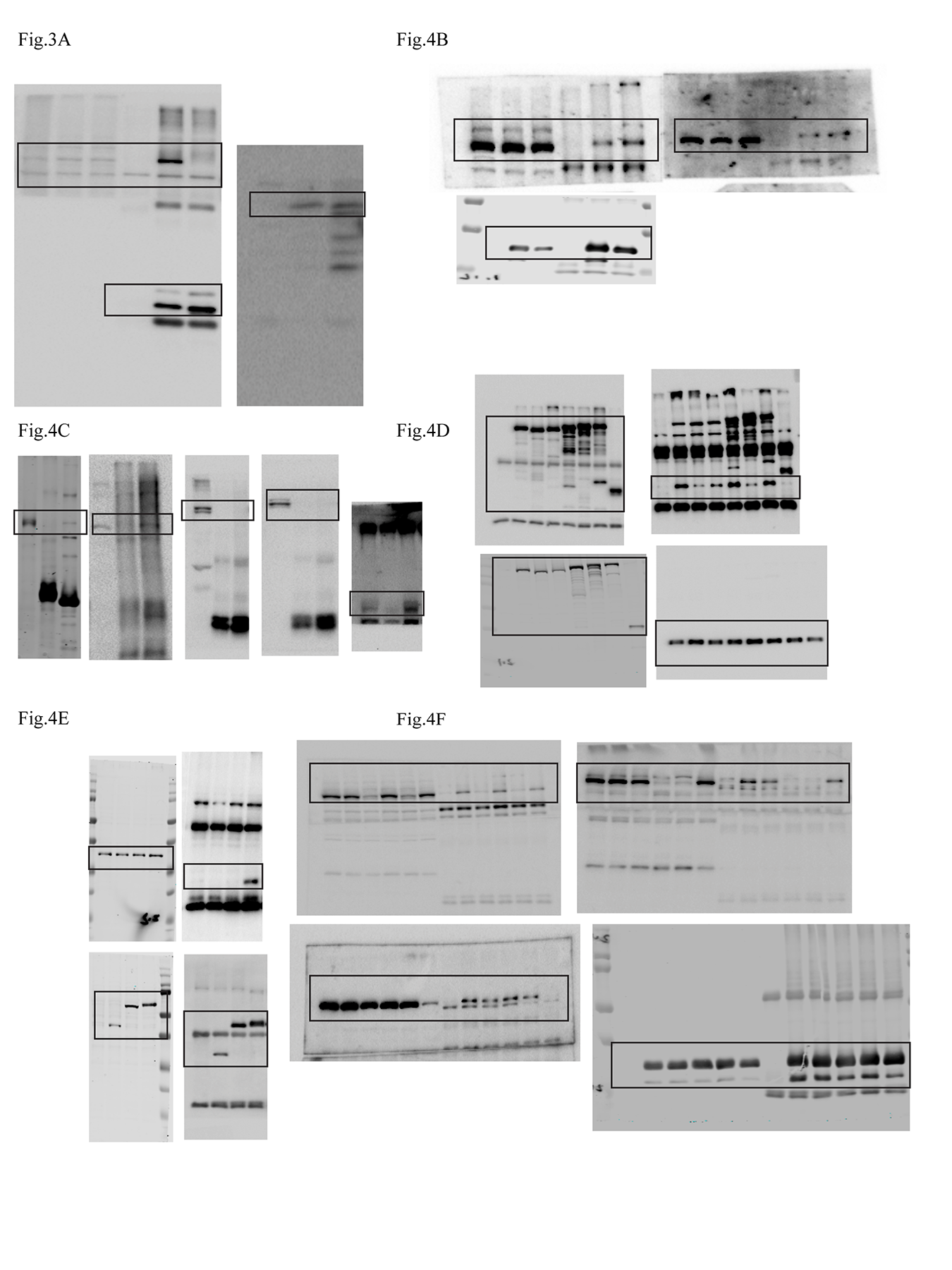

Supplement: Supplementary file 1 [file LSA-2021-01346_SdataF1.tif]

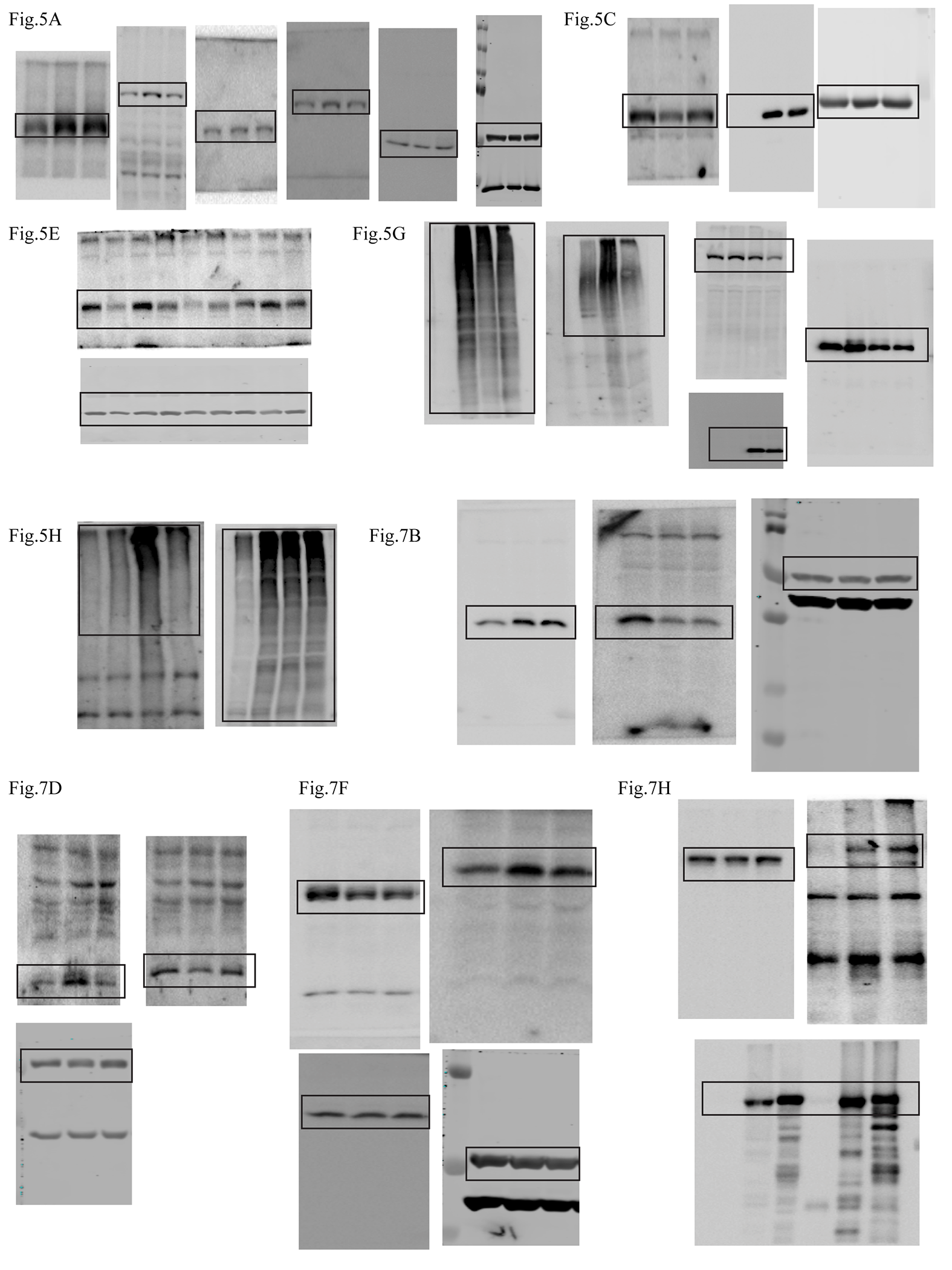

Supplement: Supplementary file 2 [file LSA-2021-01346_SdataF2.tif]
